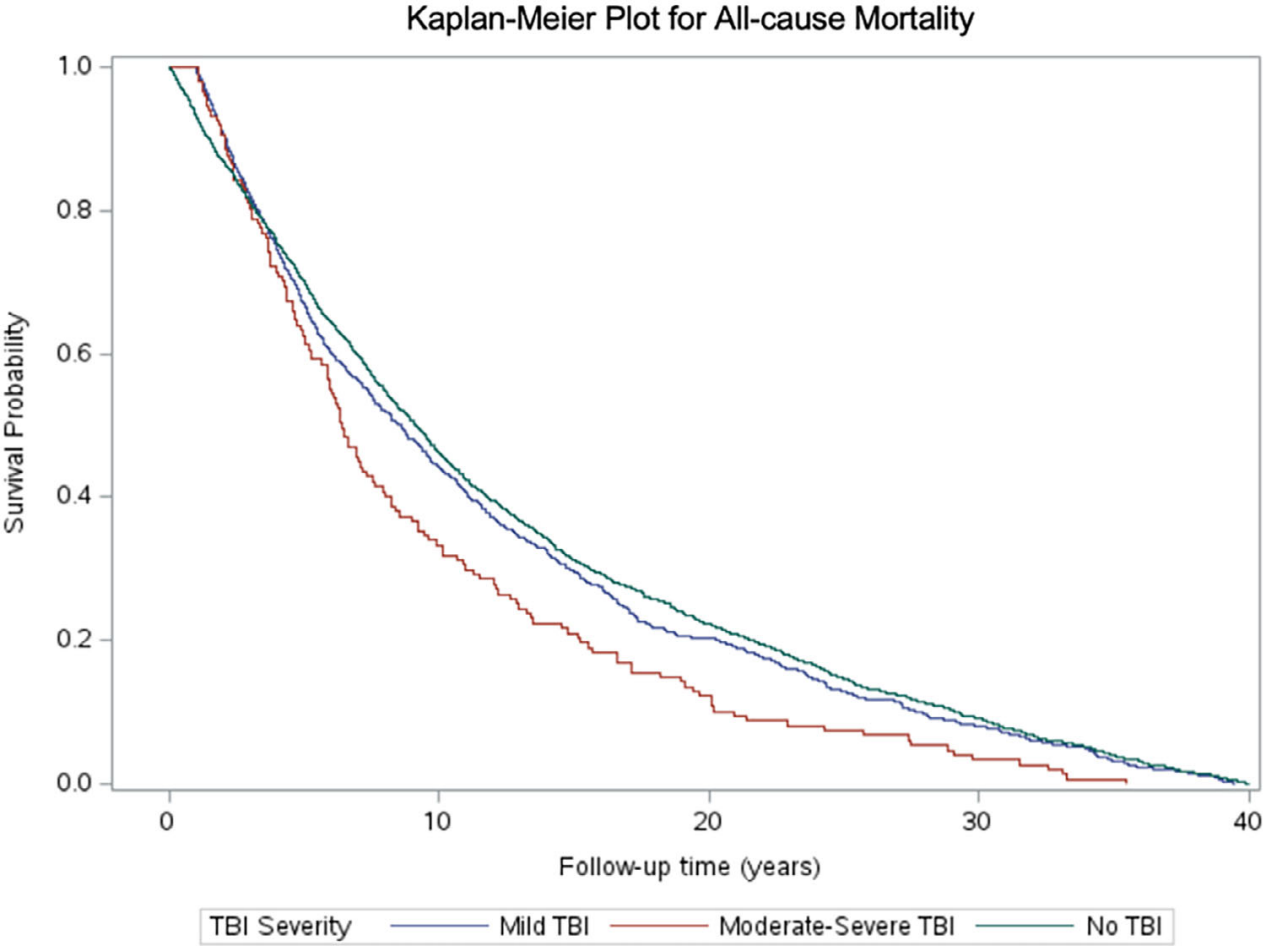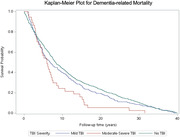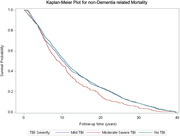# Association of Traumatic Brain Injury with All‐cause and Dementia‐related Mortality: the Framingham Heart Study

**DOI:** 10.1002/alz.092372

**Published:** 2025-01-09

**Authors:** Shruti Durape, Rebecca Burton, Prajakta S. Joshi, Kurtis Chien‐Young, Eden Price, Eukyung Yhang, Yulin Liu, Ting Fang Alvin Ang, Sherral A. Devine, Ashita S. Gurnani, Douglas I Katz, Yorghos Tripodis, Michael L. Alosco, Rhoda Au, Kristen Dams‐O'Connor, Jesse Mez

**Affiliations:** ^1^ Department of Neurology, Boston University Chobanian & Avedisian School of Medicine, Boston, MA USA; ^2^ Boston University Alzheimer’s Disease Research and CTE Centers, Boston University Chobanian & Avedisian School of Medicine, Boston, MA USA; ^3^ Framingham Heart Study, Boston University Chobanian & Avedisian School of Medicine, Boston, MA USA; ^4^ Department of Anatomy & Neurobiology, Boston University Chobanian & Avedisian School of Medicine, Boston, MA USA; ^5^ Boston University School of Public Health, Boston, MA USA; ^6^ Boston University Chobanian & Avedisian School of Medicine, Boston, MA USA; ^7^ Boston University Alzheimer’s Disease Research Center, Boston, MA USA; ^8^ Boston University Chobanian & Avedisian School of Medicine and School of Public Health, Boston, MA USA; ^9^ Icahn School of Medicine at Mount Sinai, New York, NY USA; ^10^ Framingham Heart Study, Boston, MA USA

## Abstract

**Background:**

Although traumatic brain injury (TBI) is known to be associated with short term mortality, its effects on long‐term mortality remain less clear. TBI is also a well‐known risk factor for dementia. We hypothesized that TBI would be associated with long‐term mortality, particularly dementia‐related mortality.

**Methods:**

The Framingham Heart Study (FHS) is a population‐based cohort study which recruited its original cohort between 1948‐1950, and its offspring cohort between 1971‐1975. Information on TBIs was collected across the lifespan by comprehensive review of medical records, FHS health history updates and exams and self‐report. TBI occurrence and severity were operationalized using modified American Congress of Rehabilitation Medicine and Veterans Affairs/Department of Defense criteria, respectively. Dementia diagnosis was operationalized using Diagnostic and Statistical Manual of Mental Disorders, 4^th^ Edition, and National Institute of Neurological and Communicative Diseases and Stroke/AD and Related Disorders Association criteria. Participants with history of TBI were matched to those without TBI for age and sex, with a 1:3 ratio. Participants who died within a year of TBI were excluded. Cox proportional hazards models assessed TBI risk for all‐cause mortality and cause‐specific competing risk models assessed risk for dementia‐related mortality.

**Results:**

Over the course of study, 72.82% died, with 12.32% of deaths from dementia‐related mortality. 1,688 participants with TBI (Mean age at TBI = 69 (*SD* = 15.5) years, 59.89% women) and 5,064 age‐ and sex‐matched participants without TBI were followed for an average of 15±12 years after TBI. Hazard ratios (HRs) for all‐cause mortality were 1.09 (95%CI 1.00‐1.20) and 1.66 (95%CI 1.31‐2.09 for mild and moderate‐to‐severe TBI respectively. Hazard ratios (HRs) for dementia‐related mortality were 1.63 (95%CI 1.32–2.02) and 2.67 (95%CI 1.57–4.56) for mild and moderate‐to‐severe TBI respectively. Hazard ratios (HRs) for non‐dementia related mortality were 0.96 (95%CI 0.89–1.04) and 1.15 (95%CI 0.95–1.38) for mild and moderate‐to‐severe TBI respectively.

**Conclusion:**

In a multi‐generational community‐based sample followed since the 1940s, mild and moderate‐to‐severe TBIs were associated with all‐cause mortality with the effect driven by dementia‐related mortality.